# Multifunctional roles of the autoimmune disease-associated tyrosine phosphatase PTPN22 in regulating T cell homeostasis

**DOI:** 10.1080/15384101.2015.1007018

**Published:** 2015-02-25

**Authors:** Robert J Salmond, Rebecca J Brownlie, Rose Zamoyska

**Affiliations:** Institute of Immunology and Infection Research; Ashworth Laboratories; University of Edinburgh; Edinburgh, UK

**Keywords:** autoimmunity, phosphatase, PTPN22, signal transduction, T cell

## Abstract

The non-receptor tyrosine phosphatase PTPN22 has a vital function in inhibiting antigen-receptor signaling in T cells, while polymorphisms in the *PTPN22* gene are important risk alleles in human autoimmune diseases. We recently reported that a key physiological function of PTPN22 was to prevent naïve T cell activation and effector cell responses in response to low affinity antigens. PTPN22 also has a more general role in limiting T cell receptor-induced proliferation. Here we present new data emphasizing this dual function for PTPN22 in T cells. Furthermore, we show that T cell activation modulates the expression of PTPN22 and additional inhibitory phosphatases. We discuss the implication of these findings for our understanding of the roles of PTPN22 in regulating T cell responses and in autoimmunity.

## Abbreviations

Ag: antigen
LIP: lymphopenia-induced proliferation
PTPN(22): protein tyrosine phosphatase, non-receptor type (22)
SNP: single nucleotide polymorphism
TCR: T cell antigen receptor

## Introduction

A critical feature of adaptive immunity is the ability of T cells to discriminate between benign environmental or self-antigens (Ag) and dangerous infectious agents. In order to accomplish this task, the processes of positive and negative selection in the thymus ensure central tolerance i.e., the development of a diverse peripheral T cell repertoire that expresses functional T cell receptors (TCRs) capable of recognizing foreign Ag while having low affinity for self Ag (reviewed in ^1^). Furthermore, peripheral tolerance mechanisms are also critical to prevent inappropriate activation of T cells and subsequent inflammation and autoimmunity. Such mechanisms include the specialized suppressive functions of FoxP3^+^ regulatory T cells (Tregs)^2^ and, in addition, effector T cell-intrinsic processes. The expression and function of inhibitory protein tyrosine phosphatases in TCR signaling pathways is one such cell-intrinsic mechanism by which effector T cell activation is limited.^3^

T cell receptor engagement by peptide-MHC complexes induces the phosphorylation of immunoreceptor tyrosine-based activation motifs within the intracellular tails of the TCR / CD3 complex by the src-family kinase Lck.^4^ This in turn leads to recruitment and activation of the Syk family kinase zeta chain-associated protein kinase of 70 kDa (ZAP70) and the initiation of a signaling cascade that ultimately results in the activation of gene expression, metabolic reprogramming, cell growth and division and the acquisition of specialized effector cell functions such as cytokine secretion and target cell cytotoxicity (reviewed in ^5^). Importantly, inhibitory tyrosine phosphatases, such as PTPN22, are critical both in setting the threshold for initial T cell activation and in facilitating the return of the cells to a resting state by downregulating the activation of these signaling pathways. Of particular interest we, and others, generated mice deficient in expression of PTPN22 and showed that this cytoplasmic tyrosine phosphatase is critical for maintaining T cell homeostasis.^6,7^ Furthermore, a wealth of data has accumulated in the past decade identifying *PTPN22* single-nucleotide polymorphisms (SNPs) as risk factors for the development of autoimmune diseases such as rheumatoid arthritis, type 1 diabetes and lupus (reviewed in ^8^).

Recently, we reported that PTPN22 function is important for the ability of T cells to discriminate between low affinity self-Ag and high affinity foreign Ag.^9^ In this Extra Views article, we discuss the mechanisms by which PTPN22 regulates TCR activation in response to low affinity Ag, yet also has a more general role in limiting T cell proliferation in vivo. Furthermore, we discuss how T cells regulate phosphatase expression, the overlapping and non-redundant functions of PTPN22 and other inhibitory phosphatases in T cell activation and the implications of our results for our understanding of the role of PTPN22 in autoimmunity.

## Materials and Methods

### Mice and cell transfer experiments

*Ptpn22* exon 1*^fl/fl^* × PC3-Cre, *Ptpn22* exon 1*^fl/fl^* × PC3-Cre OT-1 *Rag1^−−^* and *Ptpn22* exon 1*^fl/fl^* × dLck-Cre mice strains have been described.^6,9^ *Rag1^−−^*, CD45.1^+^, CD45.2^+^ and OT-1 CD45.1^+^ mice were bred in-house at the University of Edinburgh. For transfer experiments, bone marrow-derived dendritic cells were generated in the presence of GM-CSF, as previously described.^10^ 3 × 10^4^ WT and *Ptpn22^−−^* OT-1 T cells were co-transferred with peptide-loaded or control dendritic cells to CD45.1/CD45.2 recipient mice. In some experiments, 2.5-5 × 10^5^ sorted WT and *Ptpn22^−−^* naïve CD44^low^ CD4^+^ T cells were transferred i.v. to sublethally irradiated recipient mice. Where indicated, mice received i.p. injections of 300 μg IL-7R mAb (clone A7R34) every 48 h over the course of the experiment. In some experiments, recipient mice received ampicillin, metronidazole, neomycin sulfate and vancomycin (1 g/L) in drinking water for 10 d preceding cell transfer and throughout the course of the experiment. Flow cytometry analysis of lymph node T cells was performed using a MACSQuant flow cytometer (Miltenyi Biotech). Antibodies were from BD PharMingen, eBioscience and BioLegend. All animal procedures were licensed by the UK Home Office and performed in line with the ethical guidelines of the University of Edinburgh.

### Quantitative RT-PCR and western blotting

For RT-PCR, WT OT-1 cells were stimulated for the stated time periods with 10^−8^ M SIINFEKL (N4) peptide and total RNA prepared using Qiagen RNEasy columns. cDNA was synthesized using Superscript reverse transcriptase and quantitative PCR performed using Taqman probes (both Life Technologies). Levels of mRNA expression of phosphatase genes *Ptpn22*, *Ptpn6, Ptpn11*, *Dusp5* and *Dusp6* were normalized to expression of *rn18s*. For protein gel blotting, naïve CD44^low^ and effector-memory phenotype CD44^high^ CD8^+^ T cells were sorted by flow cytometry from lymph nodes of C57BL/6J mice and cell lysates prepared in RIPA lysis buffer. Western blots were performed using ERK2 rabbit polyclonal Abs (Santa Cruz Biotechnology) and the PTPN22 P1 Ab (a kind gift of Prof. A. Chan, Genentech).

## Results and Discussion

### Mouse models of PTPN22 function in T cell homeostasis

In order to determine the role of PTPN22 in T cell activation, we generated mice with homozygous expression of *Ptpn22* alleles with floxed exon 1.^6^ Mice were crossed with the PC3-Cre transgenic or distal (d)Lck-Cre transgenic strains in order to generate mice with ubiquitous (*Ptpn22^−−^*) or T cell-specific deletion of *Ptpn22* (*Ptpn22^fl/fl^* dLck), respectively. Of note, dLck-Cre drives deletion of floxed genomic sequences in post-selection thymocytes thus negating possible effects of gene deletion on thymocyte selection processes.^11^ In the complete absence of PTPN22, mice develop splenomegaly and lymphadenopathy as a consequence of increased numbers and frequencies of effector and effector-memory phenotype T cells and present with elevated antibody titres and increased numbers of germinal centers.^6,7,12^ Treg function and number is also elevated and is likely to be necessary to curb the highly inflammatory effector T cells present in *Ptpn22*-deficient mice, thereby preventing the induction of spontaneous autoimmunity.^6,13^ The nature of the defect in T cell homeostasis that occurs in the absence of PTPN22 was investigated by crossing PTPN22-deficient mice to an OT-1 TCR transgenic background. OT-1 T cells express an MHC Class-I restricted TCR specific for the SIINFEKL peptide derived from ovalbumin.^14^ Altered peptide ligands of SIINFEKL were used to determine the impact of PTPN22 loss on responses to Ag of varying affinities.

### Lymphopenia promotes autoreactivity that is counteracted by PTPN22

One situation, in which otherwise tolerant T cells can be driven to proliferate and gain effector function in response to self-Ag, is transient or chronic lymphopenia. Elevated levels of stromal-derived homeostatic cytokines such as IL-7 and IL-15 combine with TCR-dependent responses to low affinity and environmental Ags to drive lymphopenia-induced proliferation (LIP).^15^ Interestingly, recent evidence has shown that LIP is a primary cause of secondary autoimmunity in cohorts of multiple sclerosis (MS) patients. Thus, patients treated with alemtuzumab to deplete autoreactive lymphocytes showed relief from MS symptoms; however, a proportion of the patients suffered secondary autoimmune thyroiditis as a consequence of T cell population expansion in the lymphopenic environment.^16^

**Figure 1.**
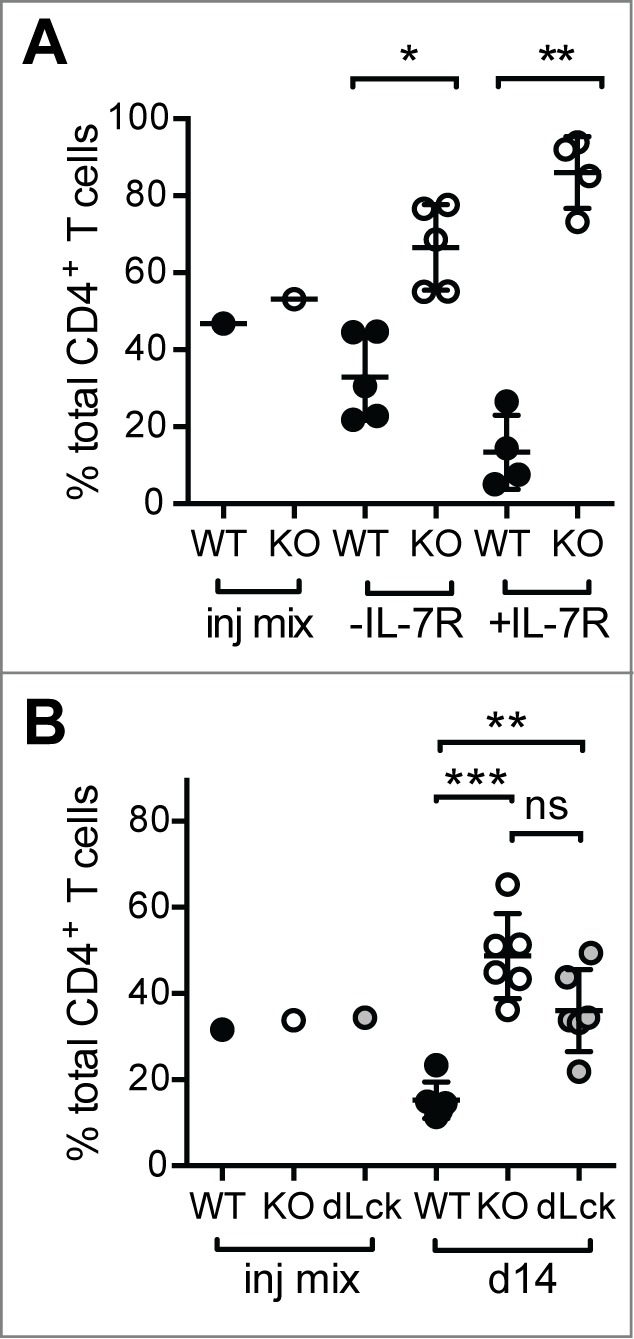
Elevated LIP of *Ptpn22^−/-^* T cells is driven by low affinity TCR signals. (**A**) IL-7R blockade exaggerates the increase in the KO:WT T cell ratio in lymphopenic hosts. Polyclonal WT and *Ptpn22^‑/−^* naïve CD4^+^ T cells were mixed ∼1:1 (injection (inj) mix) and transferred to sublethally irradiated recipient mice. Groups of mice received i.p. injections of IL-7R mAb (+ IL-7R) every second day. (**B**) Antibiotic treatment does not prevent enhanced LIP of PTPN22-deficient T cells. Naïve CD4^+^ T cells from WT, *Ptpn22^­-/−^* (KO) and *Ptpn22^fl/fl^* dLck mice were mixed ∼1:1:1 (Inj. Mix) and transferred to sublethally irradiated, antibiotic treated mice. In all cases after 14d, proportions of donor T cells in LN were assessed by flow cytometry. Lines represent mean values within groups and circles represent values from individual mice. ns – not significant, * - *P* < 0.05, ** - *P* < 0.01, *** - *P* < 0.001 by paired Students’ *t*-test.

We showed that PTPN22 is important to limit LIP as both *Ptpn22^−−^* CD8^+^ OT-1 TCR transgenic and polyclonal naïve *Ptpn22^−−^* CD4^+^ T cell populations expanded to a greater extent than their WT counterparts upon co-transfer to lymphopenic *Rag1^−−^* or sublethally irradiated recipient mice.^9^ To determine whether this was a consequence of increased responsiveness to cytokines and/or weak TCR agonists, we performed several additional experiments. Congenically marked naïve polyclonal CD4^+^ T cells from WT CD45.1^+^ and *Ptpn22^−−^* CD45.2^+^ mice were purified by FACS-sorting and co-transferred to sublethally irradiated CD45.1/CD45.2 mice that were subsequently treated with either a blocking IL-7R mAb or diluent. While IL-7R blockade reduced both WT and *Ptpn22^−−^* LIP (data not shown), the relative increase in the ratio of *Ptpn22^−−^* WT cells seen in untreated mice was exacerbated by IL-7R mAb treatment (**Fig. 1A**). Similar results were seen with CD8^+^ OT-1 TCR transgenic T cells^9^ indicating that PTPN22 is required to restrain both CD4^+^ and CD8^+^ T cell responses to TCR signals in lymphopenic conditions.

In irradiated and immunodeficient mice, polyclonal T cells populations can be driven to expand by antigens derived from enteric bacteria due to a breakdown in gut integrity.^17^ In order to limit such responses, purified naïve CD4^+^ T cells from WT CD45.1^+^, *Ptpn22^−−^* CD45.2^+^ and *Ptpn22^fl/fl^* dLck GFP^+^ mice were mixed 1:1:1 and co-transferred into sublethally irradiated host mice that were treated with an antibiotic cocktail. In antibiotic-treated recipient mice, CD4^+^ T cells from both *Ptpn22^−−^* and *Ptpn22^fl/fl^* dLck mice expanded to a significantly greater extent than WT cells (**Fig. 1B**). Importantly, there was no significant difference in the response of *Ptpn22^−−^* and *Ptpn22^fl/fl^* dLck cells. As *Ptpn22^fl/fl^* dLck cells lose expression of PTPN22 protein after positive selection, these data confirm that the increased responsiveness of *Ptpn22* knockout T cells is not likely to be the result of altered selection in the thymus. Taken together, these experiments suggest that the enhanced LIP of PTPN22-deficient T cells is driven primarily by self- rather than microbial Ags.

Further experiments confirmed that PTPN22-deficiency had a striking impact on the responses of OT-1 T cells to low affinity Ag. Thus, activation of TCR signaling pathways and induction of expression of key transcription factors and cytokines by weak agonists was markedly enhanced in the absence of PTPN22, whereas most initial responses to high affinity SIINFEKL peptide were indistinguishable from those of WT cells.^9^ Together, these data suggest that a primary function of PTPN22 in T cells is to prevent inappropriate activation of T cells by low affinity and self-Ag. This raises the important questions: are disease-associated PTPN22 variants functionally impaired in the regulation of LIP and responses to self-Ag and does this underly the role of these polymorphisms in autoimmune disease?

The disease-associated *PTPN22* 1858C→T SNP, that results in an R620W amino acid substitution, was initially described to result in a gain of function variant protein with higher catalytic activity,^18,19^ while others have argued that the polymorphism results in a loss of PTPN22 function.^20^ More recently, several groups have demonstrated that *Ptpn22* R619W knock-in mice, modeling the human SNP, have a similar, albeit milder, immunological phenotype as *Ptpn22*-deficient mice.^21,22^ Irrespective of the precise molecular consequences of the disease-associated SNPS on PTPN22 function, it may prove insightful to investigate the impact of R619W/R620W variants on LIP. For example, while *PTPN22* SNPs are not associated with susceptibility to MS, it would be of great interest to assess whether expression of these alleles influences the risks of secondary autoimmunity following alemtuzumab treatment.^16^

### PTPN22 regulates T cell proliferation

**Figure 2.**
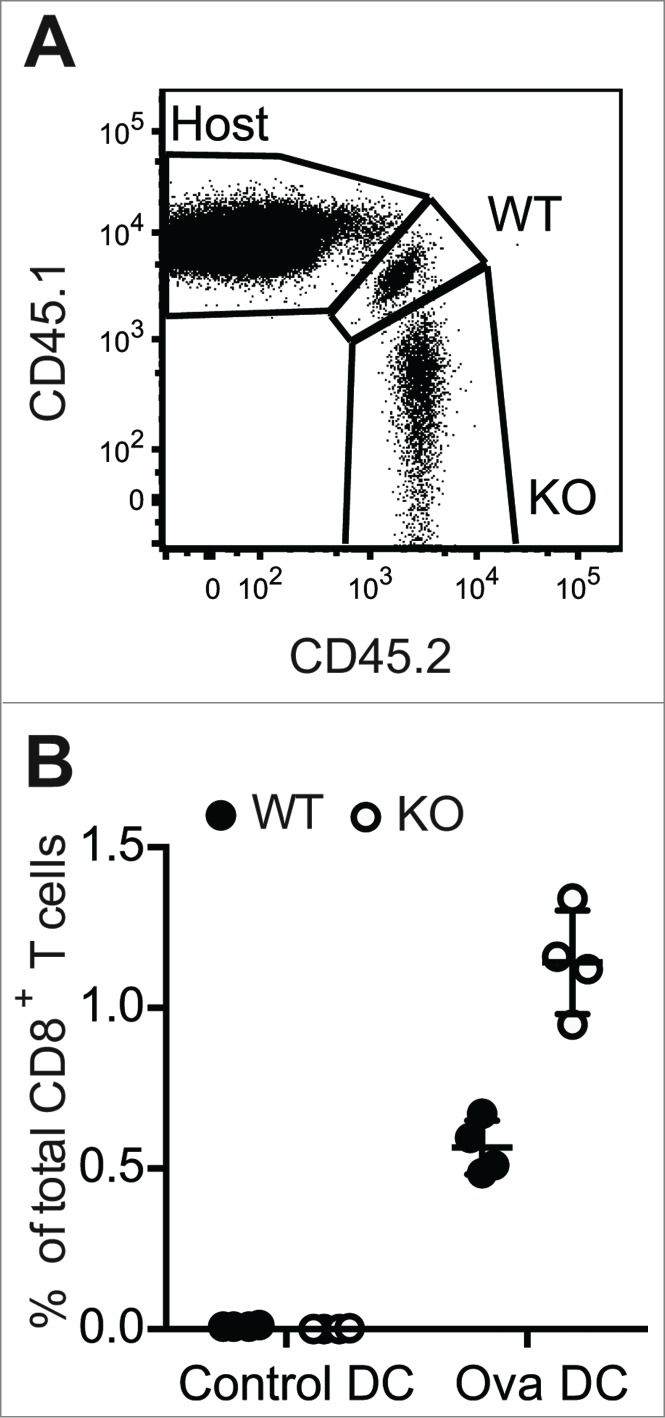
Increased expansion of *Ptpn22^‑/−^* T cell populations in response to in vivo challenge with high affinity antigen. CD45.1/CD45.2 WT and CD45.2 *Ptpn22^‑/−^* OT-1 T cells were transferred i.v. to CD45.1 recipient mice that were subsequently challenged i.v. with unpulsed (control) or ovalbumin-pulsed (Ova) DCs. After 7d, proportions of host and donor CD8^+^ T cells in LNs were assessed by flow cytometry (**A**). Lines represent mean values within groups and circles represent values from individual mice (**B**).

While our data indicate that PTPN22 is critical for regulating early TCR signals that facilitate discrimination of low affinity Ag, PTPN22 also plays a more general role in later aspects of the response, particularly in the control of TCR-induced proliferation. Previous data from our laboratory and others demonstrated that, upon stimulation with CD3/CD28 antibodies, *Ptpn22^−/−^* T cells proliferated to a greater extent than WT counterparts in vitro.^6,7^ Furthermore, we reported enhanced population expansion of *Ptpn22^−/−^* OT-1 T cells in response to SIINFEKL peptide in vitro or after transfer of the T cells into *Rag1^−/−^* mice and infection with ovalbumin-expressing *Listeria monocytogenes* (LmOva) bacteria.^9^ The lymphopenic environment of the *Rag1^−−^* recipient mice complicates the interpretation of the results from LmOva infection experiments. Therefore, to address how *Ptpn22^−/−^* OT-1 T cells behave in a T cell replete environment, WT CD45.1/CD45.2 OT-1 and *Ptpn22^−/−^* CD45.2 OT-1 T cells were mixed 1:1 and transferred to lymphocyte replete CD45.1 hosts. Recipient mice were then challenged with ovalbumin-loaded bone marrow-derived dendritic cells (Ova-DCs) or control DCs. Seven days after challenge, lymph nodes were taken from mice and the proportions of donor OT-1 T cells evaluated by flow cytometry (**Fig. 2A**). Both WT and *Ptpn22^−/−^* OT-1 cells proliferated in response to Ova-DCs, as evident by the enhanced proportions of these cells present in LNs as compared to control DC-treated mice (**Fig. 2B**). Similar to results in lymphopenic *Rag1^−/−^* environments, *Ptpn22^−/−^* OT-1 T cells were proportionally increased in comparison to WT cells in all Ova-DC recipient mice, confirming that expression of PTPN22 limits the proliferation of WT T cells to high affinity cognate Ag in vivo (**Fig. 2B**).

These data confirm that PTPN22 expression regulates the expansion of T cells to both strong and weak antigens, raising the question: how does PTPN22 limit T cell proliferation? In this regard, levels of the key transcription factor cMyc were elevated in *Ptpn22^−−^* as compared to WT OT-1 T cells following 24 h stimulation with strong agonist SIINFEKL peptide as well as in responses to weaker agonists.^9^ Importantly, cMyc has an essential role in the regulation of cell cycle progression and for the metabolic changes required for proliferation and differentiation of T cells following TCR triggering.^23^ A further, not mutually exclusive, possibility is that enhanced autocrine production of IL-2, as has been shown for *Ptpn22^−−^* polyclonal CD4^+^ T cells,^7^ drives the elevated levels of proliferation.

### Activation-induced regulation of phosphatase expression in T cells

**Figure 3.**
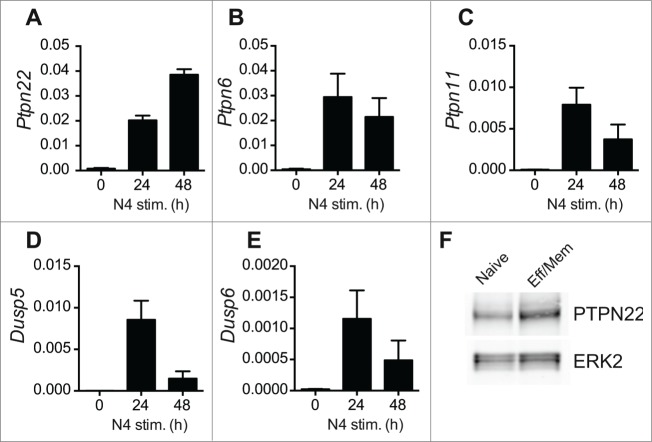
Activation-induced upregulation of phosphatase expression in OT-1 T cells. (**A-E**) Levels of individual phosphatase mRNAs in WT OT-1 cells stimulated with SIINFEKL (N4) peptide in vitro for 0–48 h were calculated by qRT-PCR. Values are normalized to expression of 18S rRNA and represent means ± SEM (n = 3). (**F**) Western blot analysis of PTPN22 expression in FACS-sorted naïve and effector-memory (Eff/Mem) phenotype polyclonal CD8^+^ T cells. ERK2 serves as a loading control. Data are representative of 2 repeated experiments.

Recent evidence has shown that T cells modulate the level of expression of inhibitory phosphatases in response to TCR triggering. For example, PTPN6 (SHP-1) expression is upregulated upon T cell stimulation and may act to counteract responses to high affinity Ags.^24^ We performed quantitative RT-PCR experiments to assess the levels of expression of PTPN22 and additional inhibitory phosphatases at various timepoints after OT-1 T cell activation. Levels of PTPN22, PTPN6, PTPN11, DUSP5 and DUSP6 mRNA were low in naïve OT-1 T cells and strongly upregulated following 24 h of stimulation with agonist SIINFEKL peptide (**Fig. 3A–E**). With the exception of PTPN22 and PTPN6, however, mRNA for the other phosphatases examined tended to decline by 48 h, by which time the T cells have entered division. We confirmed by western blotting that high levels of PTPN22 were maintained in effector/memory phenotype T cells by comparing PTPN22 abundance in naturally arising CD44^high^ T cells ex vivo with naïve T cells (**Fig. 3F**).

The expression of several tyrosine phosphatases and dual-specificity phosphatases is also downregulated in T cells in a coordinated manner via the action of micro-RNAs (miR). Specifically, miR-181a acts to enhance TCR signaling by downregulating expression of PTPN22, PTPN11, DUSP5 and DUSP6, but not PTPN6.^25^ Interestingly miR-181a is expressed at higher levels in naïve T cells, relative to effector Th1 or Th2 populations,^25^ consistent with the low level expression of its target phosphatases in naïve T cells. Together, these data imply that low level expression of PTPN22 (and possibly other phosphatases) is sufficient to limit naïve T cell activation by low affinity and self-Ags but does not curb responses to high-affinity foreign Ag. By contrast, the upregulation of PTPN22 and other inhibitory phosphatases upon TCR triggering likely acts as a safeguard to prevent excessive T cell-mediated inflammation following activation.

### Overlapping functions of tyrosine phosphatases in T cells?

Our recent data indicates that PTPN22 has a non-redundant function in controlling responses of naïve T cells in a lymphopenic environment and in response to low affinity Ag.^9^ Despite this, it is important to note that other phosphatases also function to limit LIP. T cells deficient in expression of PTPN2 (T cell-PTP) also undergo rapid proliferation upon transfer to lymphopenic hosts.^26^ Furthermore, similar to the effects of PTPN22-deficiency, the elevated LIP of *Ptpn2*^−−^ T cells is a result of elevated TCR-driven, rather than IL-7-driven, responsiveness. It is interesting to note that the expression of polymorphisms in *PTPN2* have also been associated with an enhanced risk of developing autoimmunity.^27,28^

It is possible that the similarity of phenotypes of *Ptpn2^−−^* and *Ptpn22^−−^* T cells results from shared substrate specificity. The regulation of phosphorylation of upstream kinases such as Lck is important for the promotion and termination of TCR signaling. Several phosphatases including PTPN22, PTPN2, PTPN6 and CD45 negatively regulate Lck function by modulating levels of phosphorylation of the active site Tyr394 residue.^29-33^ In each case, whether these effects are via direct dephosphorylation or are indirect has yet to be fully elucidated. Nonetheless, it is clear that several phosphatases are expressed in T cells that have shared functionality. The apparently counter-intuitive observations that PTPN2 and PTPN22 have similar targets in TCR signaling yet are not functionally redundant may be explained by their distinct patterns of expression. Thus, PTPN22 is expressed at low levels in naïve T cells (**Fig. 3**) whereas PTPN2 is expressed at elevated levels in this population and while PTPN22 expression increases upon activation the abundance of PTPN2 protein remains constant.^26^ Alternatively, it may be that there are simply insufficient quantities of either phosphatase alone to accomplish efficient regulation of TCR proximal signaling.

## Conclusions

Our recent data have provided insight into the physiological functions of PTPN22 in the regulation of T cell tolerance. Future work may unravel how these mechanisms are perturbed by SNPs and aid in our understanding of the molecular basis of T cell-mediated autoimmune disease.
